# Systematic Biopsy vs. Prostatectomy: Evaluating Correlations and Grading Discrepancies in Prostate Cancer

**DOI:** 10.7759/cureus.68075

**Published:** 2024-08-29

**Authors:** Andrada Loghin, Maria Cătălina Popelea, Ioan A Nechifor-Boilă, Angela Borda

**Affiliations:** 1 Histology, “George Emil Palade” University of Medicine, Pharmacy, Science and Technology of Targu Mures, Târgu Mureș, ROU; 2 Pathology, Mures Clinical County Hospital, Târgu Mureș, ROU; 3 Anatomy, “George Emil Palade” University of Medicine, Pharmacy, Science and Technology of Targu Mures, Târgu Mureș, ROU; 4 Urology, Mures Clinical County Hospital, Târgu Mureș, ROU; 5 Pathology, Targu-Mures Emergency County Hospital, Târgu Mureș, ROU

**Keywords:** systematic biopsy, radical prostatectomy, grade group, accuracy, prostate cancer

## Abstract

Background

Prostate Cancer (PCa) represents a growing global health challenge. The main factor in predicting PCa prognosis is represented by the Gleason Score (GS) therefore, the accuracy of pathological features from preoperative biopsy is critical in the management of the patient. We aimed to investigate the correlation between prostate biopsy parameters and the prostatectomy specimen pathological features and to identify factors that lead to over- and under-grading tumors in biopsy samples.

Materials and methods

We performed a retrospective study that included 110 male patients with confirmed PCa, selected based on specific inclusion criteria. Biopsy and radical prostatectomy (RP) specimens were analyzed using standard histopathological techniques, and pathological features were assessed according to the latest guidelines. Statistical analysis was performed using IBM SPSS Statistics version 26.0.0 (IBM Corp., Armonk, NY).

Results

The study included 110 male patients with a median age of 67 years old, ranging from 48 to 79 years old. Correlations between biopsy parameters and RP outcomes were assessed and revealed several key findings. The tumoral length on biopsy was correlated with positive surgical margin (r=0.289, p<0.01) and with tumoral volume (r=0.526, p<0.001) on prostatectomy. Patients with higher grade groups (GG) on biopsy had an approximately four times higher chance of exhibiting extraprostatic extension. We demonstrated a significant correlation between Gleason Pattern 4 (%GP4) on biopsy and pT stage, with pT4 showing the highest %GP4, and a noticeable increase in %GP4 as the pT stage progressed from pT2b to pT4. The study found a significantly higher rate of undergrading at biopsy (30.90%) compared to overgrading (6.36%). Additionally, greater tumor length and higher tumor percentages in biopsies improved grading accuracy (p<0.001).

Conclusion

Our findings suggest that systemic biopsies play a key role in predicting pathological outcomes, especially through parameters that serve as key prognostic markers. However, due to the potential of the biopsy results to be under- or overgraded, urologists should take into consideration the advantages of using repeat biopsies or additional imaging techniques to achieve a more precise diagnosis and treatment strategy.

## Introduction

Prostate cancer (PCa) ranks as the second most common cancer in men, following lung cancer, and is the fifth leading cause of death globally [1]. The global burden of PCa is expected to reach nearly 2.3 million new cases and 740,000 deaths by 2040 due to population growth and aging, making it an increasing global health concern. Risk factors include age, ethnicity, family history, and mutations in DNA-repair genes such as BRCA2 [2]. The majority of PCa have an indolent course, with a five-year survival rate of 83% in Europe and 98% in the United States [3]. However, about 20 to 30% of men treated for localized PCa will develop recurrence, with advanced stages linked to poor prognosis [4]. The increased utilization of prostate-specific antigen (PSA) testing has resulted in higher detection rates of low-risk Ca, which usually has a negligible impact on patient survival or quality of life [5].

Determining the clinical stage of PCa is crucial for initiating an appropriate treatment strategy [6]. In most medical centers, postoperative pathological grades are estimated using transrectal ultrasound (TRUS) or transperineal ultrasound (TPUS)-)-guided prostate biopsy. The histopathological features determined through prostate biopsy play an important role for patients as they guide the treatment strategies and prognosis [7].

Guidelines recommend selecting a treatment approach based on the patient's risk of disease progression. Radical prostatectomy (RP) is considered the gold standard therapeutic strategy for high-risk patients, whereas active surveillance (AS) is typically more beneficial for those with low- or very low-risk PCa [8].

AS has become a significant management strategy for patients with low-risk PCa, with various eligibility criteria such as Gleason score (GS), PSA level, clinical stage, or percentage of disease on the biopsy cores [9]. Most protocols include patients with GS 3+3=6, while some enroll biopsies with a small amount of pattern 4. Studies show that 15%-25% of these cancers are upgraded at radical prostatectomy, [10, 11] highlighting the difficulty in predicting tumors with unfavorable characteristics; therefore, the management of GS 3+4=7 PCa through active surveillance is still debated.

For patients undergoing RP, several parameters are assessed using preoperative clinical evaluations and histopathological analyses of surgical samples. These parameters encompass age, preoperative serum PSA levels, magnetic resonance imaging (MRI), tumor size, GS, Grade Group (GG), and other prognostic factors like lymphatic, venous, perineural, and seminal vesicle invasion, as well as extraprostatic extension (EPE), positive surgical margins (PSM), and lymph node metastasis [12].

The core needle biopsy (CNB) performed before surgery is the gold standard method for acquiring critical histopathological features, including the GS has a significant prognostic value and plays a key role in diagnosing and guiding treatment decisions. However, variations in Gleason patterns across different biopsy cores from the same prostate are frequent due to the heterogeneity and multifocality of the disease [13,14]. This discrepancy leads to a reduced reliability of CNB in terms of evaluating the histopathological features of a tumor [13]. Reliable assessment of the biopsy samples is crucial for determining the most suitable therapeutic strategy, avoiding unnecessary radical prostatectomy, and preventing the risks of undertreatment [8]. However, under- and overgrading prostate biopsy samples represent a common occurrence in clinical practice, and significant efforts have been made to enhance the accuracy of GS evaluation on CNB [15,16].

The GS is the main factor in predicting PCa prognosis by offering insights into the degree of cancer cell differentiation, which correlates with the cells' growth rate and potential for metastasis [17]. The accuracy of pathological features from preoperative biopsy is critical in the patients' management and the therapeutic strategy; therefore, the reliability of these results is highly significant. Errors in the biopsy technique due to the urologist or errors related to the pathologist can lead to incorrect GS evaluation, ISUP grading, and D`Amico risk group classification, resulting in under- or overtreatment. Consequently, ensuring a strong correlation between the GS from biopsy and RP is essential [17].

The use of PSA screening and CNB has led to a significant increase in PCa diagnoses, mainly because of the identification of latent low-risk tumors. Accurately differentiating between clinically significant and indolent PCa based on prostate needle biopsy results is essential for beneficial treatment [18]. Although the literature shows a strong correlation between the parameters evaluated on biopsy samples and RP specimens, the presence of inconsistent results is a matter of concern.

The main purpose of our study was to investigate the correlation between the pathological features of PCa diagnosed on biopsy and RP specimens in the Pathology Department of Mures Clinical County Hospital over the last five years. The second aim of this study was to identify factors that lead to over- and undergrading of biopsy samples in order to improve diagnostic accuracy and treatment outcomes.

## Materials and methods

In our study, we included 110 consecutive patients who met the inclusion criteria of being diagnosed with PCa in our Pathology Department via biopsy and subsequently undergoing radical prostatectomy between January 2019 and December 2023. Cases diagnosed in our department either only by biopsy or only by RP were excluded from the study, as well as patients who received hormone therapy as neoadjuvant treatment. The protocol was approved by the ethics committee of Mures Clinical County Hospital (Nr. 3923/12.04.2024), and all data were processed with the informed consent of the patients.

All cases were reviewed by two pathologists specialized in genitourinary pathology (AL, AB) who assessed standard hematoxylin-eosin-stained slides for both biopsy and RP specimens. The Gleason Score and GGs were assessed according to WHO/ISUP 2014 guidelines, and the pathological staging was established according to TNM classification. Perineural invasion was considered when tumoral cells were along or surrounding nerve fibers, and EPE was considered when tumoral cells were infiltrating the adipose tissue (on the biopsy specimens) or the prostatic capsule (on the RP specimens).

The biopsy specimens were received in 9% neutral formalin in six containers, each containing two cores from the apex, mid, and base of the right and left lobes respectively. The tissue cores were placed into cassettes for standard processing, and the H&E slides were examined by our genitourinary pathologists. Immunohistochemistry was not performed in the selected cases. The histopathological report included the number of cores sent, length of each core (measured in mm), number of cores affected by the tumor, percentage of tumor on the cores (total length of the tumor foci times 100 divided by the total length of the cores), Gleason Score, GG, cribriform pattern (if present), perineural invasion (if present), and EPE (if present).

After overnight fixation in 9% neutral formalin, the RP specimens were grossed after the following protocol: the apex, bladder neck, and seminal vesical junction were resected, sectioned perpendicularly, and completely included. A longitudinal section from each seminal vesicle was also submitted. The prostate specimen was subsequently sliced serially at 3-mm intervals, perpendicular to its long axis, from the apex to the base, and the slices were completely submitted in order to assess the tumor volume. The tumor volume was determined using Chen et al.'s formula, [19] which approximates the tumor as an ellipsoid by multiplying the length, width, and height of the tumor focus by 0.4 and 1.2, where 0.4 adjusts for the geometric fit of an irregular tumor into an ellipsoid and 1.2 is a retraction coefficient that varies between 1 (no retraction) and 1.5, depending on the laboratory. The histopathological report of the RP specimen included laterality of the tumor, Gleason score, GG, tertiary Gleason score (if present), pathological TNM stage, tumor volume, perineural invasion (if present), EPE (if present), PSM, the length of PSM (if present), and Prostate Intraepithelial Neoplasia (PIN - if present). Pathologists had access to the previous biopsy results when assessing the radical prostatectomy specimens.

Statistical analysis

Statistical analysis was performed using IBM SPSS Statistics version 26.0.0 (IBM Corp., Armonk, NY). The data distribution was assessed by examining the kurtosis and skewness as well as the histograms and QQ plots and was further validated with the Shapiro-Wilk test for normal distribution. The results confirmed a nonparametric distribution. Consequently, quantitative data was expressed as median (Q1-Q3 range), while qualitative data was expressed in absolute value (n) together with relative values (%) - n (%). Differences between groups were evaluated using the Mann-Whitney U test or Kruskal-Wallis test for quantitative data and the Chi-square test or Fisher test for qualitative data, respectively. In order to evaluate correlations between the variables, Spearman’s rank correlation coefficient (r) was used, with strong correlation defined as r≥0.7, moderate as 0.4≤r0≤0.69, and weak correlation as r≤0.39. The significance level was set at α=0.05.

## Results

The study included 110 male patients with a median age of 67 years old, ranging from 48 to 79 years old. The preoperative serum PSA level was between 0.97-56.55 ng/mL, with a median of 9.67 ng/mL. The median of the total length of the biopsy cores was 122 mm, ranging from 43 to 216 mm while the median of the length of the positive cores was 17.75 mm.

The distribution of GGs on both biopsy and RP specimens, as shown in Table 1, indicates that GG2 was the most frequently diagnosed GG (63.6% on biopsy and 66.3% on RP), while GG5 on biopsy (3.6%) and GG1 on RP (2.7%) were the least common. Perineural invasion (PNI) was the most common prognostic parameter observed in both biopsy and RP specimens. Although most tumors were bilateral, with 56.3% on biopsy and 87.2% on RP, the number of bilateral cases increased by approximately 30% from biopsy to RP.

**Table 1:** Main pathological findings on biopsy and RP specimens Bio - biopsy, RP - radical prostatectomy, GG - grade group, CP - cribriform pattern, PNI - perineural invasion, EPE - extraprostatic extension, PIN - prostatic intraepithelial neoplasia, PSM - positive surgical margin

	Right PCa, n (%)	Left PCa, n (%)	Bilateral PCa, n (%)	GG1, n (%)	GG2, n (%)	GG3, n (%)	GG4, n (%)	GG 5, n (%)	CP, n (%)	PNI, n (%)	EPE, n (%)	PIN, n (%)	Multiple tumoral foci, n (%)	PSM
Bio	20 (18.1)	28 (25.4)	62 (56.3)	16 (14.5)	70 (63.6)	13 (11.8)	7 (6.3)	4 (3.6)	28 (25.4)	36 (32.7)	6 (5.4)	9 (8.1)	-	-
PR	7 (6.3)	8 (7.2)	96 (87.2)	3 (2.7)	73 (66.3)	20 (18.1)	4 (3.6)	11 (10)	-	76 (69)	54 (49)	28 (25.4)	61 (55.4)	43 (39)

Correlations between biopsy and RP parameters were assessed and the results are shown in Table 2. Serum PSA levels demonstrated a weak yet significant positive correlation with the percentage of Gleason 4 pattern (%G4) on prostatectomy specimens, as well as with tumoral volume. The number of positive cores showed a moderate, significant positive correlation with tumoral volume and a weak, significant positive correlation with the presence of the PSM. Positive tumoral length and tumor percentage on biopsy were weakly positively correlated with the PSM and moderately correlated with tumoral volume on prostatectomy. Additionally, the %G4 on biopsy showed a weak, positive correlation with the tumoral volume. 

**Table 2:** Correlations between biopsy parameters and prostatectomy outcomes PSM - positive surgical margin r - Spearman correlation coefficient

	Percentage of Gleason 4 pattern on prostatectomy specimen		Tumoral volume on prostatectomy specimen		PSM	
	r	p	r	p	r	p
Prebiopsy serum PSA level	0.247	<0.01	0.288	<0.01	0.179	0.06
Number of positive cores	-0.22	0.82	0.532	<0.001	0.241	0.01
Positive tumoral length on biopsy	0.095	0.32	0.526	<0.001	0.289	<0.01
Tumor percentage on biopsy	0.093	0.33	0.547	<0.001	0.293	<0.01
Percentage of Gleason 4 pattern on biopsy specimen	0.380	<0.001	0.239	<0.01	0.044	0.64

Associations between higher aggressivity GG (≥3) identified on biopsy specimens and RP prognostic factors were examined, and the results are shown in Table 3. Patients with GG ≥3 on biopsy had an approximately four times higher chance of exhibiting EPE, while there was no significant association between GG ≥3 and the presence of PSM.

**Table 3:** Associations between GGs on biopsy specimens and RP prognostic factors RP - radical prostatectomy, GG - grade group **Chi-square test

	GG<3 on biopsy, n (%)	GG ≥3 on biopsy, n (%)	p**	OR (CI95%)
Extraprostatic extension, n (%)	36 (41.9)	18 (75)	<0.01	4.167 (1.505-11.537)
Positive surgical margin, n (%)	31 (36)	12 (50)	0.21	1.774 (0.712-4.422)

Table 4 shows the correlation between the presence of PNI on biopsy specimens and RP prognostic factors such as GG, PSM, pT stage, EPE, and tumoral volume. The presence of perineural invasion on biopsy specimens correlated weak but statistically significant with GGs, pT stage, EPE, and tumoral volume, but the correlation with PSM was not statistically significant.

**Table 4:** Correlation between PNI on biopsy and RP prognostic factors PNI - PNI - perineural invasion, RP - radical prostatectomy r - Spearman correlation coefficient

	GG on RP			PSM			pT stage			Extraprostatic extension			Tumoral volume		
	r		p	r	p		r		p	r		p	r		p
PNI on biopsy specimen	0.27	0.0031		0.03		0.7	0.34	0.0002		0.28	0.002		0.24	0.009	

**Figure 1:**
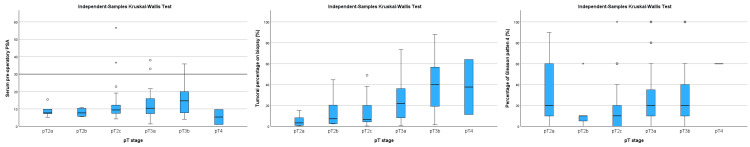
Correlations between pT stage and serum pre-operatory PSA, tumoral percentage on biopsy, and percentage of Gleason pattern 4 pT - pathological, PSA - prostate-specific antigen

The correlations between preoperative parameters and pathological stage are shown in Table 5 and illustrated in Figure 1. Patients with an advanced pT stage exhibited a significantly greater positive length on biopsy (p<0.001). Moreover, the percentage of tumoral tissue on biopsy correlated with the pathological stage, with pT3b and pT4 stages showing the highest tumoral percentage, whereas pT2a demonstrated the lowest value (p<0.01). The variations in the percentage of Gleason pattern 4 (%GP4) on biopsy were also notable, with the pT4 stage presenting the highest percentage and a rising trend observed from pT2b to pT4 stages (p=0.01).

**Table 5:** Correlation between pre-operative parameters and pathological stage (pT) *Kruskal Wallis test

	pT2a, n=5	pT2b, n=6	pT2c, n=44	pT3a, n=32	pT3b, n=21	pT4, n=2	p*
Age (years)	67 (59-73)	62 (55-69)	68 (63-72)	66 (64-69)	65 (62-70)	62 (60-65)	0.34
Serum PSA (ng/mL)	7.77 (6.24-12.57)	7.72 (5.72-10.44)	9.32 (7.32-12.27)	10.42 (7.32-15.97)	14.70 (7.77-20.50)	5.26 (0.97-9.56)	0.10
Total positive length on biopsy (cm)	5.00 (1.00-15.50)	7.00 (2.00-24.25)	9.25 (4.50-20.87)	26.50 (8.62-51.75)	48.00 (20.50-68.50)	48.50 (17.00-80.00)	<0.001
Tumoral percentage on biopsy (%)	3.18 (0.62-11.67)	7.18 (2.56-26.29)	6.63 (4.08-20.01)	21.74 (8.14-41.47)	39.75 (17.82-57.68)	37.70 (11.40-64.00)	<0.001
Percentage of Gleason pattern 4 on biopsy (%)	20.00 (5.00-75.00)	10.00 (3.75-22.50)	10.00 (0.00-20.00)	20.00 (10.00-37.50)	20.00 (7.50-50.00)	60.00 (60.00-60.00)	0.01

The correlations between prognostic parameters on biopsy and the pT stage are presented in Table 6. Patients with advanced pT staging (pT2c-pT4) were significantly more likely to present higher GGs diagnosed on biopsy (GG ≥3), while those with pT2a or pT2b stages were more often associated with lower GG (<3). Moreover, patients with advanced pT stages (pT2c-pT4) demonstrated a statistically significant increasing trend in the presence of bilateral adenocarcinoma (p<0.001), perineural invasion (p<0.01), and EPE (p<0.01).

**Table 6:** Correlations between prognostic parameters on biopsy and pT stage pT - pathological **Chi-square test

	pT2a, n=5 (%)	pT2b, n=6 (%)	pT2c, n=44 (%)	pT3a, n=32 (%)	pT3b, n=21 (%)	pT4, n=2 (%)	p**
Grade group (GG)							
GG1, n (%)	1 (20)	1 (16.7)	12 (27.3)	2 (6.3)	0 (0)	0 (0)	<0.001
GG2, n (%)	2 (40)	4 (66.7)	29 (65.9)	24 (75)	11 (52.4)	0 (0)	
GG3, n (%)	2 (40)	1 (16.7)	2 (4.5)	3 (9.4)	3 (14.3)	2 (100)	
GG4, n (%)	0 (0)	0 (0)	1 (2.3)	3 (9.4)	3 (14.3)	0 (0)	
GG5, n (%)	0 (0)	0 (0)	0 (0)	0 (0)	4 (19)	0 (0)	
Bilateral adenocarcinoma, n (%)	0 (0)	0 (0)	23 (52.3)	21 (65.6)	16 (76.2)	2 (100)	<0.001
Cribriform pattern, n (%)	0 (0)	2 (33.3)	6 (13.6)	13 (40.6)	6 (28.6)	1 (50)	0.08
PIN, n (%)	1 (20)	0 (0)	3 (6.8)	4 (12.5)	1 (4.8)	0 (0)	0.72
Perineural invasion, n (%)	2 (40)	1 (16.7)	6 (13.6)	14 (43.8)	11 (52.4)	2 (100)	<0.01
Extraprostate extension, n (%)	0 (0)	0 (0)	0 (0)	1 (3.1)	5 (23.8)	0 (0)	<0.01

In order to assess the diagnostic accuracy of CNB and evaluate the potential of under- or overgrading, the discrepancies between GG at biopsy and RP were analyzed using contingency tables and a marginal homogeneity test. The marginal homogeneity test yielded statistically significant results, with a p-value of <0.001. This highlights a significant difference in the distribution of GG categories between the two diagnostic points, suggesting that both overgrading and undergrading occur. The contingency table analysis (Table 7) demonstrated a significantly higher incidence of undergrading, with 34 cases (30.90%), compared to overgrading which was present in only seven cases (6.36%).

**Table 7:** The incidence of under-/overgrading PCa PCa - Prostate cancer

bGG/RPGG	GG1, n (%)	GG2, n (%)	GG3, n (%)	GG4, n (%)	GG5, n (%)	p
GG1, n (%)	3 (100)	12 (16.4)	1 (5.3)	0 (0)	0 (0)	<0.001
GG2, n (%)	0 (0)	55 (75.3)	13 (68.4)	1 (25)	1 (9.1)	
GG3, n (%)	0 (0)	6 (8.2)	4 (21.1)	0 (0)	3 (27.3)	
GG4, n (%)	0 (0)	0 (0)	1 (5.3)	3 (75)	3 (27.3)	
GG5, n (%)	0 (0)	0 (0)	0 (0)	0 (0)	4 (36.4)	

Moreover, out of the 16 patients who were diagnosed with GG1 on biopsy and underwent RP, 13 were over-graded on the RP specimen, while three received the same diagnosis. Comparing these two categories of patients (those over-graded from GG1 to GG2 and those with GG1 on both biopsy and RP), we observed significant differences in the average biopsy sample size (126 mm vs. 140 mm), average percentage of tumors on biopsy (7.42% compared to 0.59%) as well as tumor laterality (46.1% of bilateral tumors in the over-graded group compared to 33.3% in the GG1 group). Regarding PSA levels, no differences were observed between the two groups (average 8.10 ng/mL versus 8.89 ng/mL). On the RP specimen, the only notable difference was the average tumor volume was 1 cc and 0.2 cc, respectively.

We examined how biopsy length and percentage of tumor may affect the accuracy of grade grouping and the results are shown in Table 8. The analysis demonstrated that patients with greater tumor length and higher tumor percentage on biopsy were more likely to be accurately graded. This finding emphasizes that grading accuracy is substantially influenced by the length of the tumor and its percentage on the biopsy cores. There was no statistically significant correlation between the PSA value and the accuracy of the grading.

**Table 8:** The association between biopsy parameters and grading accuracy ***Mann-Whitney U test

	Correctly graded	Incorrectly graded	p***
Tumor length on biopsy (cm)	28 (5.75-52.75)	7.75 (4.62-25.12)	<0.001
% of tumor on biopsy (%)	28 (6.5-52.5)	7.75 (5-25)	<0.001
PSA value (ng/mL)	9.7 (10.9-15.6)	9.6 (8.5-11.6)	0.2

## Discussion

PSA is secreted by prostate epithelial cells and therefore represents an organ-specific biomarker. Since the Food and Drug Administration's (FDA) approval three decades ago, PSA has been an essential tool in the diagnostic algorithm and therapeutic management of PCa. However, PSA levels may be affected by benign conditions like benign prostatic hyperplasia (BPH) or prostatitis. Despite ongoing debates over PSA screening, its levels remain a significant factor in risk assessment and prognosis at the time of PCa diagnosis [20]. In our study, the serum PSA level was not associated with the pathological stage of the tumor nor the GG, but it was significantly associated with the %GP4 on both the biopsy sample and RP specimen and also with the tumoral volume.

Accurate grading on biopsy samples is crucial because it ensures that patients suitable for active surveillance can avoid radical prostatectomy and its associated side effects, while those with high-grade cancer receive appropriate treatment, preventing the progression to advanced or metastatic disease. However, inaccurate grading of PCa is a well-known occurrence, probably because of the sampling errors and because of the tumors’ heterogeneity and multifocal nature [13]. A meta-analysis of 16 studies [15] that included approximately 15.000 patients showed an overall accuracy of 63%. Undergrading was observed in 30% of cases, ranging from 6% to 36%, while overgrading occurred in 7% of cases. Another meta-analysis that included almost 3.000 cases demonstrated an accuracy rate of 60% [21]. A recent study [22] revealed a grading concordance in 61.4% of cases, while the over- and undergrading were in 7.4% and 31.2%, respectively. Our study's results align with the existing literature, showing a 62.7% accuracy rate between biopsy and radical prostatectomy results. The present study revealed a significantly higher incidence of undergrading at biopsy (30.90%), compared to overgrading, which occurred in seven cases (6.36%). Inaccurate grading of PCa has important implications, undergrading may lead to neurovascular bundle preservation while overgrading may lead to an aggressive treatment such as radiotherapy. Consequently, there is a crucial necessity for prediction models. The risk factors for upgrading the Gleason score in PCa were studied and the existing literature shows that the length of the biopsy cores together with parameters like PSA, tumoral volume, and the percentage of the tumor on the biopsy cores can predict upgrading [10]. Our findings align with the data in the literature; the patients that have been upgraded to a higher GG presented a higher length of the biopsy samples, a higher percentage of tumor on the biopsy cores, and higher tumoral volume, compared to the patients that have been accurately graded. However, in our study, PSA values did not correlate statistically significantly with the accuracy of the grading.

Moreover, our study demonstrated that greater tumor length and higher tumor percentages on biopsies greatly improved grading accuracy, indicating that these factors significantly affect grading precision. The percentage of tumors on biopsy samples was also significantly associated with advanced pathological stage, %GP4, tumoral volume, and the length of PSM, underscoring once again the importance of the quality of biopsy sampling. The literature indicates that certain factors, including larger prostate volume, a small number of biopsy cores, a lower percentage of tumors, and shorter biopsy core lengths, have been recognized as risk factors for pathological upgrading [23]. However, in a study conducted by Fiorentino et al. [1] that included patients with fusion biopsies, the percentage of undergrading was significantly lower (17.4% vs. 30.9%), proving that fusion biopsy is potentially more accurate than systematic biopsy. Given that GS is a key factor in determining whether to pursue active surveillance or treatment, improving the accuracy of GS evaluation could have significant clinical implications.

The present study concluded that there is a significant association between the %GP4 and the pT stage, with the pT4 stage presenting the highest %GP4 on the biopsy specimen, showing a rising trend from pT2b to pT4 stages. Our results align with a study conducted by Ordner et al. [24] that assessed the relevance of the %GP4 on biopsy. The authors demonstrated that there is an association between the percentage of GP4 on the biopsy specimens and a higher GG (≥3) or an advanced pathological stage.

Perineural invasion is defined by tumoral cells invading the perineural space, entering an environment that may offer signals promoting their survival. The presence of PNI indicates the potential for the tumor to spread via lymphatic or vascular routes, which could suggest a poorer prognosis [25]. Lubig et al. [26] demonstrated that the prevalence of PNI in PCa is high, being present in 25% of biopsy samples and 75% of RP specimens. In our cohort, PNI was found on 32.7% of biopsy samples and 69% of RP specimens, aligning with data from the literature. Different countries have varying guidelines on whether identifying perineural invasion should be a required part of the pathological evaluation for PCa cases [27]. While some research indicates that PNI is linked to an increased likelihood of prostatic capsule invasion or lymphatic spread [26,28], other studies have found that PNI is not a strong predictor of prognosis for PC [27]. Moreover, there is inconsistency in how PNI is detected, with some research suggesting that its identification might be enhanced using immunohistochemistry [26]. In our study, PNI on biopsy samples was significantly associated with negative pathological features such as advanced GG, pathological stage, EPE, and tumor volume but was not significantly correlated with the risk of PSM. Moreover, 70% of patients with PNI on biopsy presented EPE on RP, demonstrating that PNI is a very important predictor of EPE and, by extension, of advanced pathological stage. Celik et al. [29] demonstrated that the presence of perineural invasion on the biopsy specimen is strongly associated with a PSM on RP. Furthermore, two systematic reviews [28,30] showed that besides the correlation between PNI and PSM, there is a strong association between PNI on biopsy and biochemical recurrence after surgical intervention. Our results showed no association between PNI and PSM, contrary to the data described in the literature, but this finding may be due to the small number of patients included in the study that may have biased the results.

This study is a retrospective, single-institution analysis, which could introduce potential bias. Additionally, the study's limitations are represented by a small patient cohort, the exclusion of patients with metastatic disease or those who received neoadjuvant therapy, and the absence of follow-up.

## Conclusions

The pathologists’ precision is essential for the accurate diagnosis and staging of malignant tumors, especially for the therapeutic management of the patient. Our findings, supported by existing literature, demonstrate that systematic biopsy plays a significant role in predicting pathological outcomes, particularly through parameters that serve as prognostic markers. Given the potential for biopsy results to be undergraded, relying exclusively on these results for treatment planning may lead to inaccuracies. We recommend that urologists address this limitation by integrating MRI-guided biopsies based on the patient's particularities, as transrectal biopsy alone without prior MRI has limited sensitivity and specificity. To validate our findings, larger prospective cohort studies with extended follow-up will be necessary.
